# Utilizing artificial intelligence to solving time – cost – quality trade-off problem

**DOI:** 10.1038/s41598-022-24668-7

**Published:** 2022-11-22

**Authors:** Pham Vu Hong Son, Luu Ngoc Quynh Khoi

**Affiliations:** Construction Engineering & Management Department, Ho Chi Minh City University of Technology (HCMUT), Vietnam National University, 268 Ly Thuong Kiet Street, District 10, Ho Chi Minh City, 700000 Vietnam

**Keywords:** Civil engineering, Computational science

## Abstract

This study presents the Slime Mold Algorithm (SMA) to solve the time—cost—quality trade-off problem in a construction project. The proposed SMA is a flexible and efficient algorithm in exploration and exploitation to reach the best optimal solution to process the input model’s data. This paper aims to discuss and solve the optimization problem and compare the evaluation with other algorithms such as Opposition-based Multiple Objective Differential Evolution, Non-dominated sorting genetic algorithm, Multiple objective particle swarm optimization, Multiple objective differential evolution and Chaotic initialized multiple objective differential evolution (CAMODE) to verify the efficiency and potential of the proposed algorithm. According to the analysis results, the SMA model generated a diversification measure for case studies, producing superior outcomes to those of previous algorithms.

## Introduction

Many disciplines, including daily life and construction management in particular, have seen significant transformation as a result of artificial intelligence. Researchers have steadily introduced new sorts of algorithms to use in balancing optimization aspects in building to increase management efficiency. This problem involves the assumption that all activities of a project can be performed in different ways in terms of cost, time, and quality. The objectives related to these three factors contradict each other, thus rendering the TCQT problem a challenging task^1–4^.

In the twenty-first century, several new algorithm chains have been developed for solving complex problems under real conditions, especially in the construction industry. The continued development of novel algorithms is aimed at obtaining optimal solutions to existing problems. Several new algorithms are based on various types of algorithms. The SMA is based on biological factors and on the characteristic interactions among them. The SMA, when applied to optimize a process, balances the trade-off between cost, time, and quality and helps achieve the most optimal results, thus yielding a more competitive product.

Several studies have established various algorithms and measures for solving the TCQT problem. Some studies have developed algorithms comprising optimal combinations of construction methods for all activities^5–7^. Analytical approaches involve using mathematical methods such as linear and dynamic programming to solve problems^8–10^. However, metaheuristic approaches have been demonstrated to be more effective in solving optimization problems and used hybrid evolutionary algorithms to solve a time–quality trade-off problem^11^. Development modeled a general-priority multimethod TCQT scheduling problem by using mixed-integer mathematical programming^4^. Construction material-based outlined a new two-step methodology for solving the TCQT problem^12^. New methodologies analyzed a stochastic time–cost balancing problem by using a fuzzy memetic optimization algorithm^6^.

The SMA model is proposed to solve many problems in many different fields, especially in this study it is applied to multi-objective optimization in the construction industry. Considering the optimal ability of the model compared with many previous models has been proposed to demonstrate the efficiency and superiority of the algorithm. In the case, if it solves three factors well at the same time, it will continue to upgrade in solving more factors or will find the limitations of the model to combine with many other methods to increase the integration and show better results for this model. The main function that the SMA uses most effectively is in the exploration and then exploitation phase to be able to achieve the most optimal solution in the capabilities. The results that the proposed model brings are very positive and superior to similar models, so the author proposes the SMA model to solve optimization problems in the field of construction management.

On the basis of the preceding problems and algorithms reported in the literature, the present study utilized the SMA to obtain favorable conditions for simulating the TCQT problem. The objective of the study was to demonstrate that the SMA can achieve rapid convergence without losing diversity and help solve the TCQT problem. The SMA was compared with OMODE, NSGA-II, MOPSO and MODE algorithms to demonstrate its effectiveness. The following sections show the SMA model in process and demonstrations.

## Literature review

A novel algorithm model that is referred to as the slime mold algorithm (SMA)^13^. X-ray chest image segmentation issues were resolved using the SMA model along with Whale optimization^14^. To address the complex optimization issue, a hybrid SMA with differential evolution approach is presented^15^. The SMA has not been applied to the popular in construction management, especially to optimize important construction goals to help project managers easily select the best factors in construction projects.

Starting from this problem, many authors have performed two-factor optimization simultaneously^16,17^ and then argue that time—cost are two important factors in construction^18^. For the complete and optimal analysis of multiple objectives, the balance of time, cost and quality is essential in project implementation to achieve good results. The schedule-cost trade-off is extended to time–cost-quality^19^ and time–cost-safety trade-off optimization models^20^. The issue of time, money, and quality was resolved using the GA model^21^. Furthermore, many evolutionary hybridization algorithms have been successfully used to solve time–cost-quality^22,23^.

Although, previous studies on solving problems of optimizing time–cost-quality have made many contributions in providing improved models related to goals; However, the proposed models have many limitations such as: (1) Not all optimal cases have been considered (2) Only applied to simple construction projects (3). Many assumptions are still set. necessary for problem (4) The results for Pareto are not really good. Therefore, this study proposes to use the SMA model to solve the details more comprehensive and successfully to demonstrate the superiority of an algorithm and no longer become more difficult to implement for this type of optimization. The most special thing is to deploy the model applied to the field of construction management more to enhance the role of the algorithm for the construction industry in general.

## Methodology

The development of the SMA is based on the modification of slime mold approach behavior to illustrate exploration and exploitation ability, increase the chance of finding optimal results^13^. Applying of this function is an important property to optimize and carefully select the best candidate solutions to facilitate a good Pareto solution.

### Inspiration for the SMA

**Figure 1:**
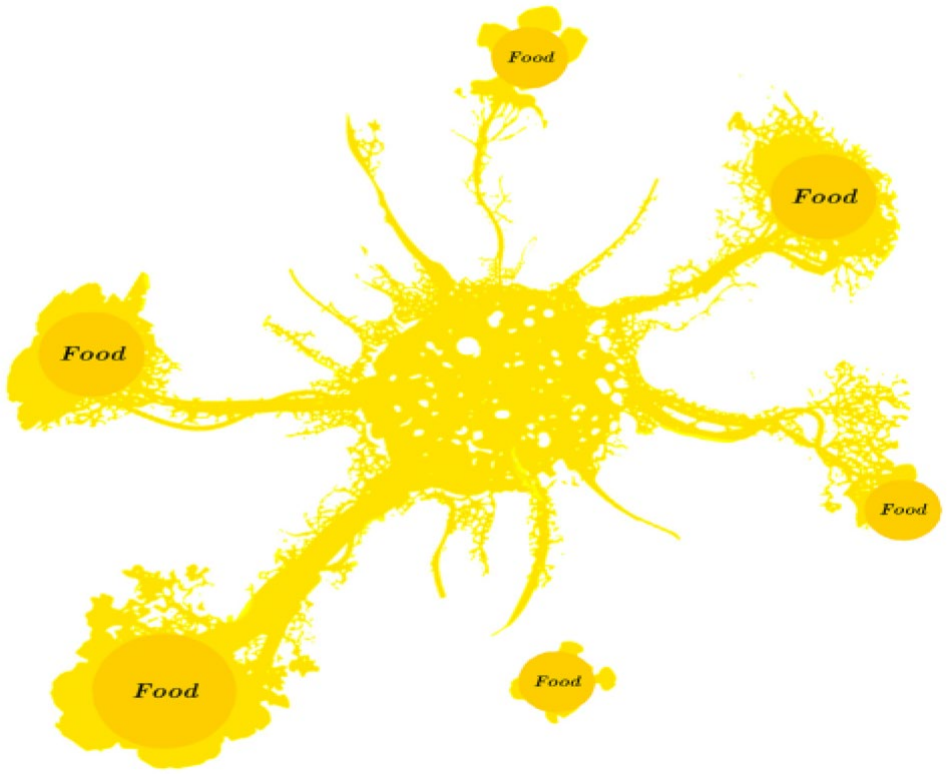
Food-finding pattern of P. polycephalum.

The life cycle of Physarum polycephalum begins with the growth of individual cells, each with its own nucleus. The cells fuse to form plasmodium, a single cell containing millions or even billions of nuclei swimming in cytoplasmic fluid. This is the vegetative phase of the protozoan’s life, during which it obtains food, surrounds itself, and secretes enzymes to digest food. This is also the time when P. polycephalum exhibits curious behavior: it can remember locations where it previously found food and can share memories with other parts of the slime mold. Such characteristics are implausible for an organism that does not possess a brain or a nervous system in Fig. 1.

### Mathematical model of the SMA

*P. polycephalum* can dynamically adjust its search patterns according to the quality of food provenance. When the quality of food sources is high, the slime mold uses a region-limited search method^24^, meaning that it focuses the search on the discovered food sources. If the density of the initially found food provenance is low, the slime mold leaves the food source to explore other alternative food sources in the region^25^. The mathematical model and procedure of the described processes are detailed in this section.

#### Approaching food

**Figure 2:**
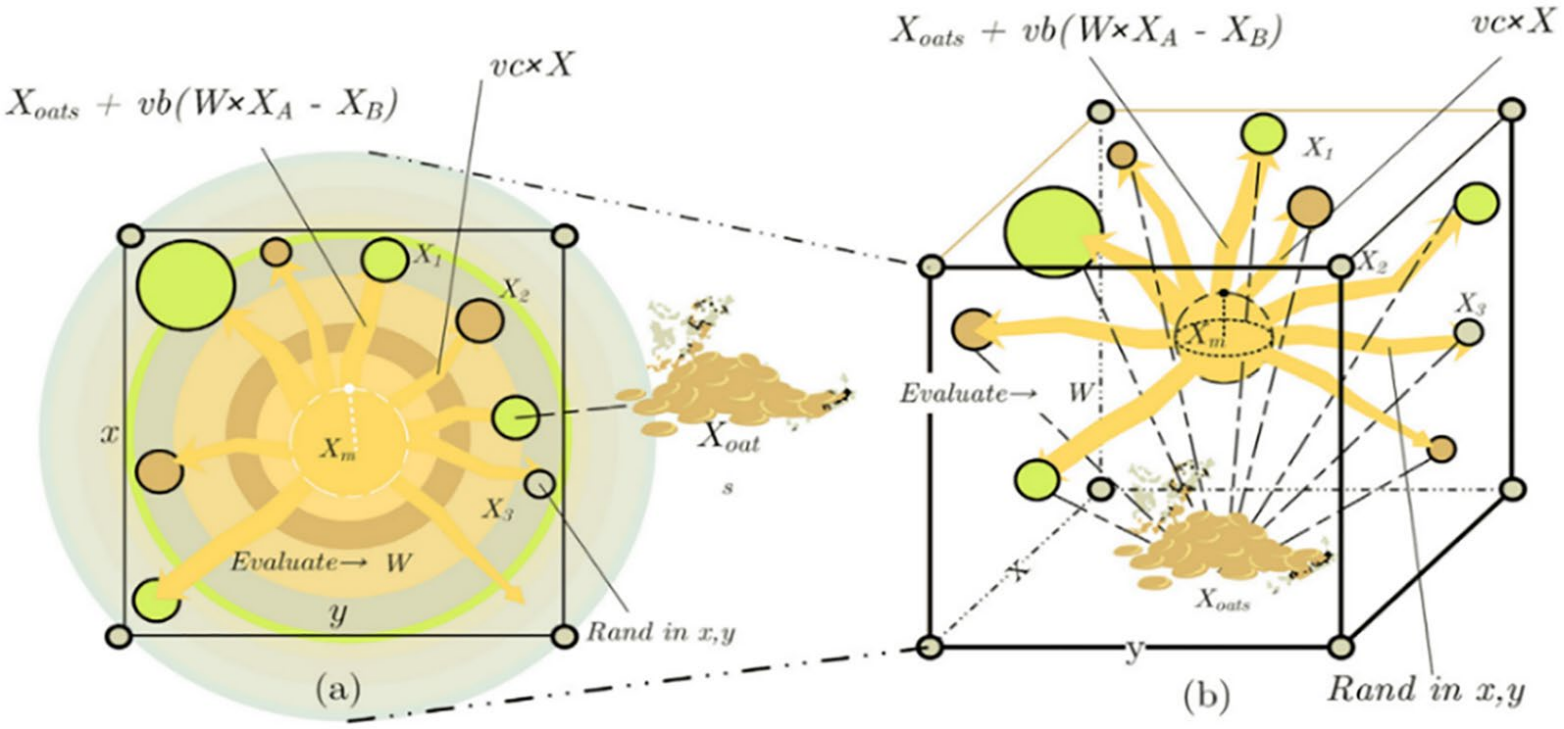
Position in the two-dimensional and three-dimensional spaces.

The slime mold can approach food by sensing odor in the air in Fig. 2. To mathematically express this approach behavior, we propose the following formulas to simulate the contraction mode:

1 $$\overrightarrow {X(t + 1)} = \left\{ \begin{gathered} \overrightarrow {{X_{b} (t)}} + \overrightarrow {vb} .(\overrightarrow {{\text{W}}} .\overrightarrow {{X_{A} (t)}} - \overrightarrow {{X_{B} (t)}} ),\,r < p \hfill \\ \overrightarrow {vc} .\overrightarrow {X(t)} ,\,r \ge p \hfill \\ \end{gathered} \right.$$

where $\overrightarrow {vb}$ is a parameter within the range [− a, a], $\overrightarrow {vc}$ is a parameter that decreases linearly from 1 to 0, *t* is the current iteration, $\overrightarrow {{X_{b} }}$ is the currently found individual location with the highest odor concentration, $\overrightarrow {X}$ is the location of the slime mold, $\overrightarrow {{X_{A} }}$ and $\overrightarrow {{X_{B} }}$ are two randomly selected individual locations of the slime mold, $\overrightarrow {{\text{W}}}$ is the weight of the slime mold, and *p* is a parameter that is expressed as follows:

2 $$p = \tanh \left| {S(i) - DF} \right|$$

**Figure 3:**
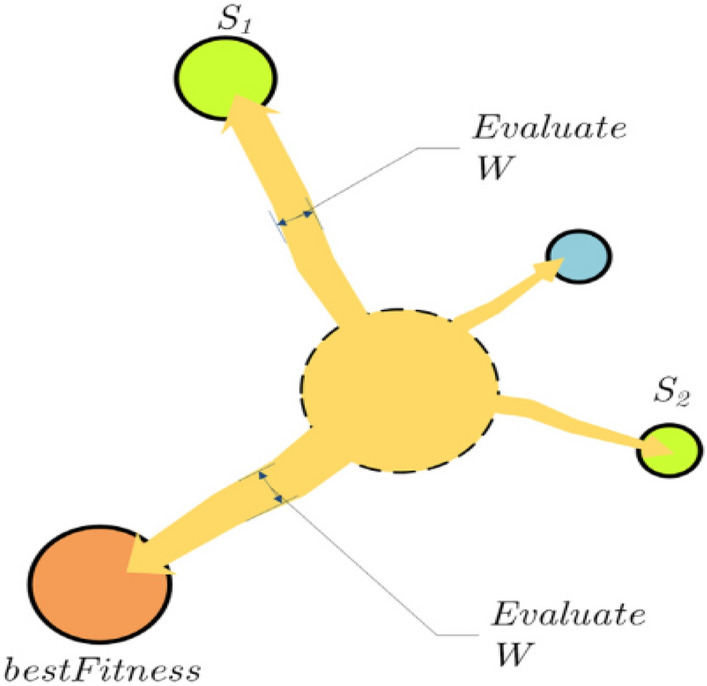
Evaluation of reasonability.

$i \in 1,\,2,...,\,n,\,S\,(i)$ is the fitness of $\overrightarrow {{\text{X}}}$.

DF is the optimal fitness obtained in all iterations.

The formula for $\overrightarrow {vb}$ is as follows:

3 $$\overrightarrow {vb} \, = \,\left[ { - \,{\text{a}},{\text{ a}}} \right]$$

The formula for *a* is as follows:

4 $$a = \arctan h( - (\frac{t}{\max \_t}) + 1)$$

The formula for $\overrightarrow {{\text{W}}}$ is as follows:

5 $$\overrightarrow {{\text{W(SmellIndex(i))}}} = \left\{ \begin{gathered} 1 + r.\log (\frac{bF - S(i)}{{bF - wF}} + 1),\,condition \hfill \\ 1 - r.\log (\frac{bF - S(i)}{{bF - wF}} + 1),\,others \hfill \\ \end{gathered} \right.$$

6 $$SmellIndex = Sort(S)$$

where *S(i)* is rank of the first half of the population, *r* is a random value within the interval [0*,* 1] *Max_t* is the maximum iteration, *bF* is the optimal fitness obtained in the current iteration, *wF* is the worst fitness value obtained in the current iteration, and *SmellIndex* is the sequence of sorted fitness values.

#### Wrapping food

On the basis of the aforementioned principle in Fig. 3, the location update mechanism of the slime mold can be expressed mathematically as follows:

7 $$\overrightarrow {{X^{*} }} = \left\{ \begin{gathered} rand.(UB - LB) + LB,\,rand < z \hfill \\ \overrightarrow {{X_{b} (t)}} + \overrightarrow {vb} .({\text{W}}.\overrightarrow {{X_{A} (t)}} - \overrightarrow {{X_{B} (t)}} ,\,r < p \hfill \\ \overrightarrow {vc} .\overrightarrow {X(t)} ,\,r \ge p \hfill \\ \end{gathered} \right.$$

where UB and LB denote the lower and upper boundaries of the search range, respectively; R and r denote random values within the range [0, 1]; and z denotes a parameter, which is described subsequently.

#### Oscillations

**Figure 4:**
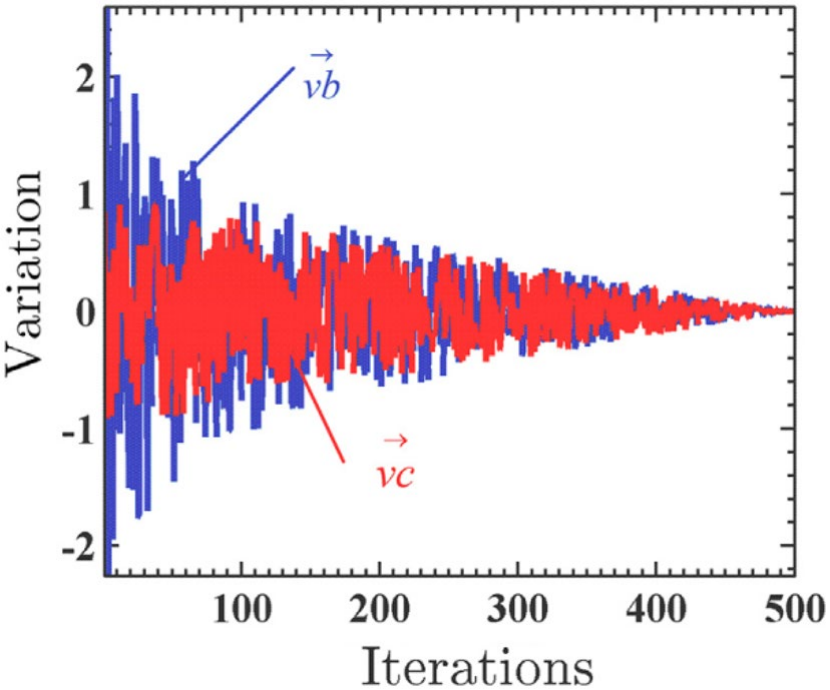
Direction of $\overrightarrow {vb}$ and $\overrightarrow {vc}$.

The slime mold primarily utilizes a propagation wave produced through biological oscillations to change cytoplasmic flow in the veins, thereby improving the position of food concentration. To simulate the variations in the venous width of the slime mold, $\overrightarrow {{\text{W}}}$, $\overrightarrow {vb}$, and $\overrightarrow {vc}$ can be used, where $\overrightarrow {{\text{W}}}$ represents the oscillation frequency of the slime mold at different food concentrations, $\overrightarrow {vb}$ represents a parameter that oscillates randomly within [− a, a] and gradually approaches zero with the increase in the number of iterations, and $\overrightarrow {vc}$ represents a parameter that oscillates within [− 1, 1] and approaches zero. Through the produced oscillations, the slime mold can approach food more quickly when it finds high-quality food; however, when the food concentration is lower at a particular position, it approaches the food slowly. This process thus improves the efficiency of the slime mold in selecting the optimal food source in Fig. 4.

#### Pseudocode of the SMA

The SMA pseudocode is shown in Table 1 and still more mechanisms can be added to the algorithm, or a more comprehensive simulation of the slime mold life cycle.

**Table 1:**
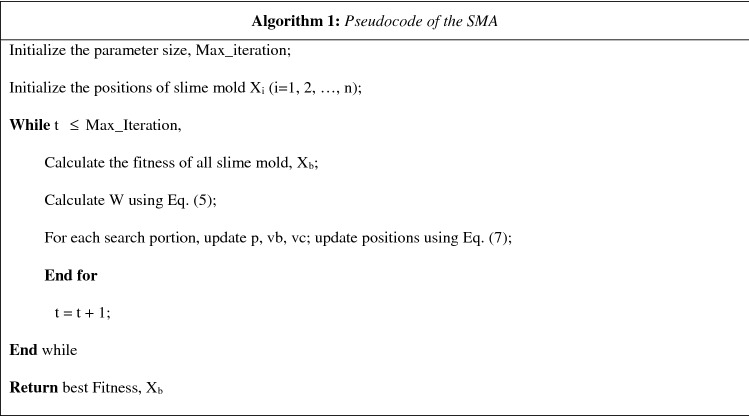
SMA Procedure.

## Using the SMA to optimize project time, cost, and quality

**Figure 5:**
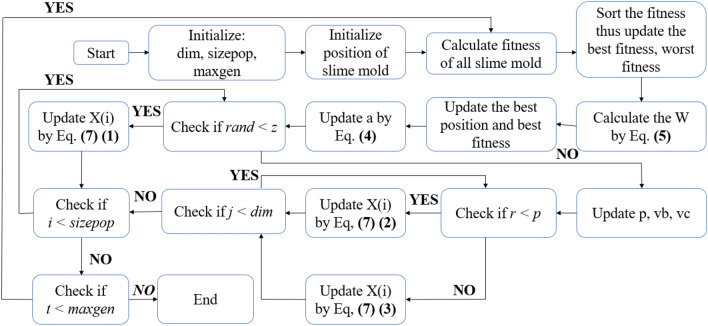
Flow of the SMA.

**Figure 6:**
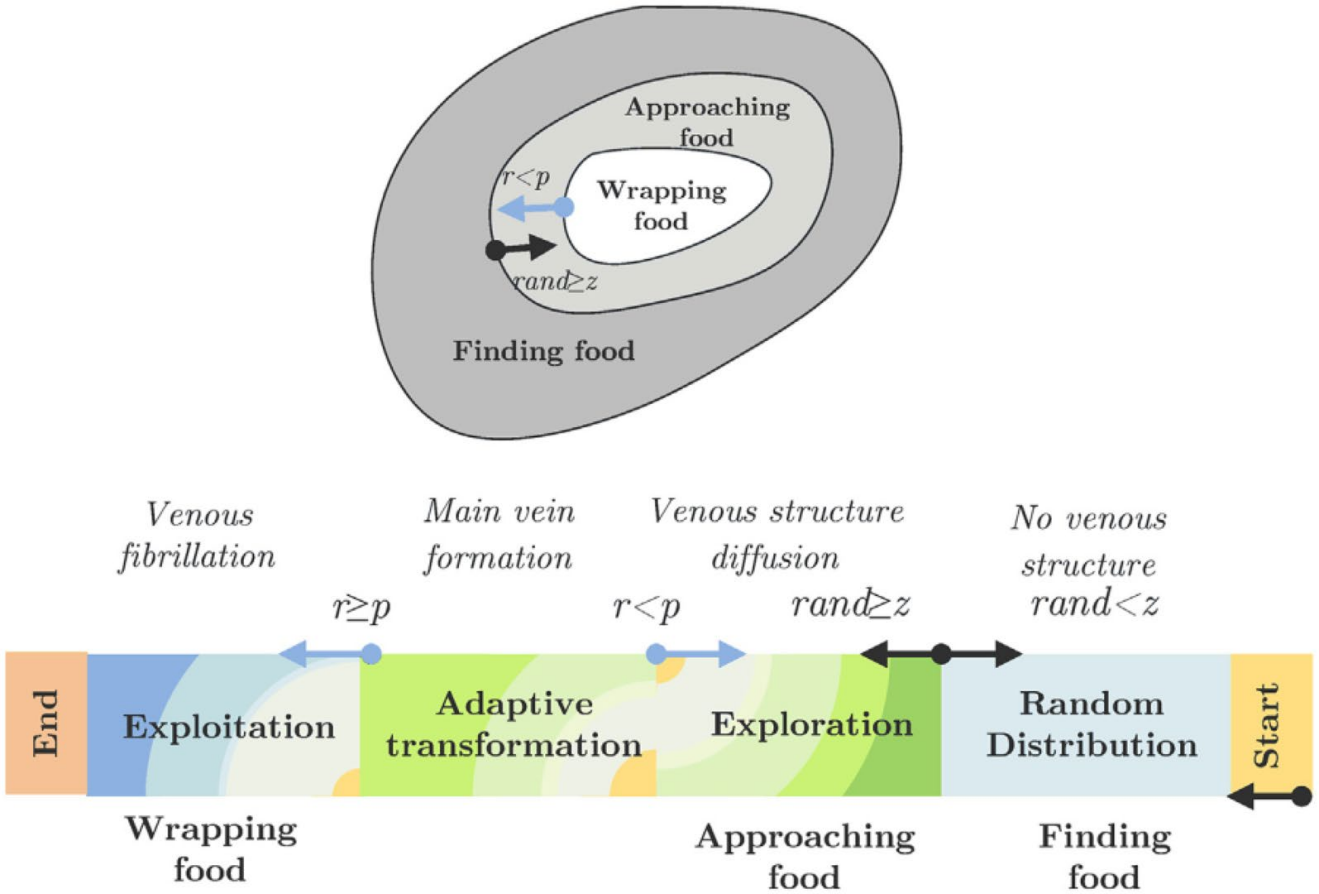
Steps in the SMA.

In this section, the SMA is presented in detail. SMA is the core optimization algorithm in the optimization model of time—cost—quality. Previous studies have never seen the SMA model applied to the field of construction management, so the development based on this model is completely new and proposed^13^. The optimal model is shown in Figs. 5 and 6.

### Declare parameters and initialize the population

This study is applied to the three factors of time, cost and quality in a construction project to be optimized simultaneously. Therefore, the input parameters of the necessary model are project-specific information such as the relationships in the works. Specifically, it is necessary to determine the number of populations, the maximum number of iterations, the minimum value LB and the maximum value UB of the variables. With the stated parameters, the optimization algorithm will automatically calculate the optimal solution for all three factors at the same time.

### Process of SMA

After initial population initialization, at each iteration, the SMA applied the model's characteristics and features to explore and exploit in the research. Declare the locations of slime molds so that the suitability of each location can be assessed. Then, calculate the features of the SMA to be able to find the best odor locations and update the new location of the slime mold.

During the optimization process, the number of populations remains constant. Therefore, populations are selected from combinations. For a single-objective optimization algorithm, the optimal solution is the solution to the objective function with the best value. However, in a multi-objective algorithm, a method must be used to solve three objectives simultaneously.

### Stop conditions

The optimization process ends when the stopping condition is satisfied. A commonly used stopping condition is the maximum number of iterations or the number of evaluations of the objective function. In the proposed model, the author uses the maximum number of iterations. When the stopping condition of the algorithm is satisfied, the optimal solutions will be given.

## Case study

This section describes the environment created to deal with time–cost-quality optimization in construction management, i.e., establishing a database created by SMA with input data of construction factors to identify the pareto solution generated from the output data. This research intends to offer information that project managers can utilize to optimize project issues and increase the effectiveness of construction investment. As a result, the author chose to apply SMA to the case study in order to replicate data processing and use it for model testing.

This study analyzed the findings reported to demonstrate the superiority of the SMA in solving the TCQT problem^26,27^. The results obtained using the SMA were compared with those obtained using the OMODE, NSGA-II, MOPSO, MODE and CAMODE algorithms. The OMODE algorithm is based on adversarial-based learning techniques that considerably improve the diversity of an original set and evolve the algorithm's diversity and convergence balancing process. The NSGA-II algorithm is highly effective in solving the TCQT problem^28^. Moreover, the MOPSO algorithm is a highly competitive swarm intelligence algorithm for goal optimization problems and is especially effective in solving project management problems^29,30^. The MODE algorithm has been extensively used in various fields to solve problems, especially in the construction field^31^. In traditional DE, the CAMODE method generates an initial set by taking advantage of chaotic series, and it employs an external elite repository to hold non-dominated solutions^27^.

For the evaluation of the mentioned algorithms, the present study focused on an actual highway construction project^19^. The project manager determined the construction time of the project and was responsible for selecting the option to be implemented. This project involved 18 construction activities, each of which involved several cases. The following scenario illustrates a construction project for a warehouse that was expanded upon from a prior project^27^. The project is divided into 37 activities, each with a unique set of instances. To make the project more complex, the author of this study suggested adding Quality criteria.

**Figure 7:**
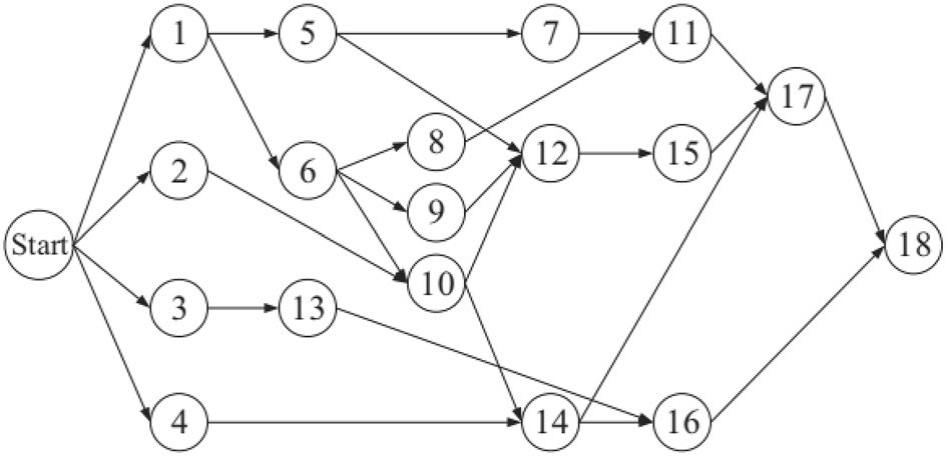
Project network for case 1.

**Figure 8:**
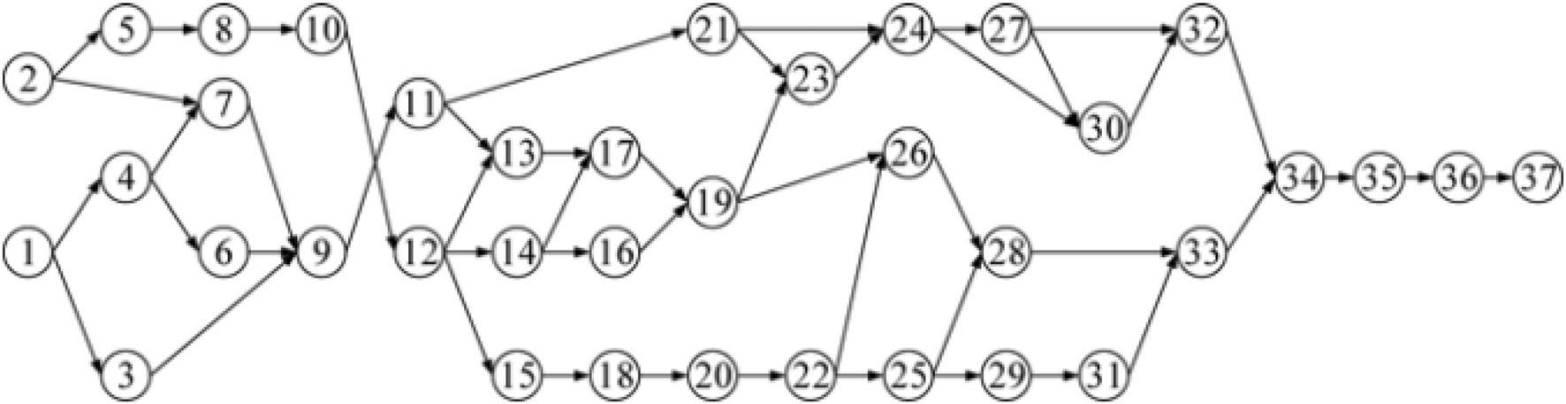
Project network for case 2.

Selecting options with low time requirements would require high costs (and vice versa). Therefore, selecting an option that would minimize the time and cost required by the project while achieving the maximum quality was necessary. The SMA was used to determine the optimal solution to these problems and thus demonstrate its effectiveness and potential. Figures 7, 8 displays the priority order of the project activities, and Table 2, 3 presents the necessary data related to time, cost, and quality for each specific activity in each specific case. To evaluate the two problems, MATLAB was used to implement the original SMA to analyze random cases in order to determine which cases would lead to a reduction in time and cost and improvement in quality. Balancing between time, cost, and quality could ensure high project performance. Accordingly, the project manager must select an option to achieve balanced optimization and thus obtain the potential solution to the problems, which can positively influence the outcome of the project. This section describes the optimization results obtained by the SMA and the findings of a previous study^26^.

**Table 2:** Resource utilization options of the project 1.

Activities	Case	Time	Cost	Weight	Ql k = 1		Ql k = 2		Ql k = 3		Quality
(i)	(n)	dates	Dollars	(%) (wt_i_)	IW (wt_i,_)	QP (Q^n^_i,_)	IW (wt_i,_)	QP (Q^n^_i,k_)	IW (wt_i,k_)	QP (Q^n^_i,k_)	
1	1	14	2400	3	50	100	30	96	20	98	2.952
	2	15	2150			90		89		89	2.685
	3	16	1900			86		77		84	2.487
	4	21	1500			75		72		73	2.211
	5	24	1200			63		60		65	1.875
2	1	15	3000	5	40	98	40	94	20	99	4.83
	2	18	2400			87		94		95	4.57
	3	20	1800			81		92		85	4.31
	4	23	1500			77		72		70	3.68
	5	25	1000			60		66		59	3.11
3	1	15	4500	8	70	100	15	97	15	95	7.904
	2	22	4000			80		82		81	6.436
	3	33	3200			62		60		63	4.948
4	1	12	45,000	11	50	99	35	95	15	94	10.6535
	2	16	35,000			74		71		76	8.0575
	3	20	30,000			59		63		64	6.7265
5	1	22	20,000	10	60	100	20	97	20	99	9.92
	2	24	17,500			93		89		89	9.14
	3	28	15,000			77		71		72	7.48
	4	30	10,000			61		64		61	6.16
6	1	14	40,000	11	50	95	25	95	25	100	10.5875
	2	18	32,000			76		74		79	8.3875
	3	24	18,000			59		62		68	6.82
7	1	9	30,000	10	30	97	30	99	40	93	9.6
	2	15	24,000			70		73		71	7.13
	3	18	22,000			61		62		67	6.37
8	1	14	220	1	100	95	0	NA	0	NA	0.95
	2	15	215			83					0.83
	3	16	200			75					0.75
	4	21	208			68					0.68
	5	24	120			61					0.61
9	1	15	300	1	50	100	50	99	0	NA	0.995
	2	18	240			97		92			0.945
	3	20	180			81		88			0.845
	4	23	150			71		75			0.73
	5	25	100			63		64			0.635
10	1	15	450	1	60	94	40	97	0	NA	0.952
	2	22	400			79		83			0.806
	3	33	320			63		69			0.654
11	1	12	450	2	70	96	30	95	0	NA	1.914
	2	16	350			72		75			1.458
	3	20	300			61		66			1.25
12	1	22	2000	3	50	99	35	98	15	95	2.9415
	2	24	1750			89		85		87	2.619
	3	28	1500			70		71		79	2.151
	4	30	1000			62		61		63	1.854
13	1	14	4000	7	40	99	40	96	20	97	6.818
	2	18	3200			73		71		76	5.096
	3	24	1800			60		62		63	4.298
14	1	9	3000	6	80	100	10	95	10	98	5.958
	2	15	2400			79		82		81	4.77
	3	18	2200			63		67		66	3.822
15	1	16	3500	7	70	100	30	98	0	NA	6.958
16	1	20	3000	3	30	97	30	96	40	98	2.913
	2	22	2000			89		85		87	2.61
	3	24	1750			81		79		78	2.376
	4	28	1500			72		73		74	2.193
	5	30	1000			67		60		62	1.887
17	1	14	4000	6	70	98	20	97	10	99	5.874
	2	18	3200			73		75		72	4.398
	3	24	1800			62		65		61	3.75
18	1	9	3000	5	30	98	45	99	25	94	4.8725
	2	15	2400			75		77		71	3.745
	3	18	2200			63		66		67	3.26752

**Table 3:** Resource utilization options of the project 2.

Act	EM	Predeccessor	D (Days)	C($)	Quality (%)
1	1	–	25	5000	2.6573
2	1	–	11	2200	2.6491
3	1	1	21	8400	2.4782
	2		16	9600	2.2222
4	1	1	20	10,000	2.7322
	2		18	10,800	2.0966
5	1	2	15	3000	2.2923
6	1	4	9	2700	2.5773
	2		6	3000	1.8189
7	1	2,4	14	5600	2.2845
	2		10	6000	2.0161
8	1	5	10	5000	2.6077
9	1	3,6,7	12	6000	2.0486
	2		11	6600	2.5707
10	1	8	10	4000	1.9724
11	1	9	12	6000	2.4940
12	1	4	10	4000	2.6715
13	1	11,12	10	5000	1.4687
14	1	12	14	7000	2.3730
15	1	12	7	2800	1.1516
16	1	14	7	2100	2.6214
17	1	13,14	7	2800	1.5583
18	1	15	12	4800	2.5968
	2		9	5400	1.9235
19	1	16,17	10	4000	2.5091
20	1	18	14	5600	2.9954
	2		10	6000	1.4442
21	1	11	7	2800	2.1764
22	1	20	14	4200	2.6299
	2		10	5000	2.5315
23	1	21,19	10	4000	1.5513
24	1	21	9	4500	2.0664
25	1	22	7	3500	1.4958
26	1	19,22	14	5600	2.4133
27	1	24	16	8000	1.1595
28	1	25,26	10	4000	2.6853
	2		9	4500	2.0508
29	1	25	6	1200	1.6109
30	1	24,27	3	900	1.1427
31	1	29	12	4800	2.0623
32	1	27,3	5	1000	1.4740
33	1	28,31	10	2000	2.0783
34	1	32,33	7	700	1.5162
35	1	34	14	700	1.9674
36	1	35	14	1400	2.0079
37	1	36	1	100	1.7801

Table 4 shows details of the input parameters for the proposed project, in order to optimize with each best results in terms of time, cost, and quality.

**Table 4:** Input parameters for case 1, 2.

Input	Notation	Value (Case 1/Case 2)
Population size	N	100/100
Maximum generation	T	500/80
Number of decision variables	D	25/25
z parameter	Z	0.03/0.03
Lower boundary	LB	−100/−100
Upper boundary	UB	100/100

### Input parameters

#### Decision variable

- The minimum total project duration can be expressed as follows:
  8 $$\begin{gathered} {\text{Minimum total project duration }} = \sum\limits_{{i = 1}}^{l} {T_{i} = Max(ES_{i} + d_{i} )} \hfill \\ ES_{i} = \mathop {Maximum(ES_{j} + d_{j} )}\limits_{{all\,predecessors\,j\,of\,i}} \hfill \\ \end{gathered}$$
  where *T*_*i*_ is the duration of the *i*th activity {*i* = 1; 2; …; l}, *l* is the total number of critical activities on a specific critical path, *ES*_*i*_ is the earliest start of the *i*th activity, and *d*_*i*_ is the duration of the *i*th activity.

- The minimum total project cost can be expressed as follows:
  9 $${\text{Minimum total project cost}}\, = \,\sum\limits_{i = 1}^{n} {\cos t_{i} }$$
  where *Cos t*_*i*_ is the cost of the *i*th activity for a specific option of execution methods and *n* is the total number of activities.

- The maximum total project quality can be expressed as follows:
  10 $${\text{Maximum total project quality}}\, = \,\sum\limits_{i = 1}^{l} {{\text{w}}t_{i} } \sum\limits_{k = 1}^{K} {{\text{w}}t_{i,k} xQ_{i,k}^{n} }$$
  where *Q*^*n*^_*ik*_ represents the performance of the quality indicator* k* in the *i*th activity using resource *n*, *wt*_*i,k*_ represents the weight of the quality indicator *k* compared with other indicators in the *i*th activity, and *wt*_*i*_ denotes the weight of the *i*th activity compared with other activities in the project.

### Optimization results obtained using the SMA

Table 5 presents the time, cost, and quality optimization results obtained using the SMA. These three factors were simultaneously optimized to obtain the optimal solution. A project manager must determine the optimal path that is both short and convenient. Such optimization can be realized through practical experience regarding specific situations in construction projects.

**Table 5:** Optimal results for case 1.

Solutions	Partial set	Optimal resource utilization options	Project performance			Gant	Iteration (/500)
			Time (Days)	Cost (Dollars)	Quality (%)		
1	Minimum total project duration	1 3 1 1 1 1 1 1 1 1 1 1 1 1 1 2 1 1	104	166,620	96.77	0 1 6 9 10 12 15 17 18	7
2		1 1 1 1 2 1 1 1 1 1 1 1 1 1 1 3 1 1	104	165,070	96.28	0 1 6 9 10 12 15 17 18	26
3		1 3 1 1 2 1 1 1 1 1 1 1 1 1 1 3 1 1	104	163,870	95.76	0 1 6 9 10 12 15 17 18	11
4	Minimum total project cost	5 5 3 3 4 3 3 5 5 2 3 4 3 3 1 5 3 3	161	99,820	65.15	1 6 9 12 15 17 18	155
5		5 5 3 3 4 3 3 5 4 2 3 4 3 3 1 5 3 3	159	99,870	65.24	1 6 9 12 15 17 18	106
6		5 5 3 3 4 3 3 5 3 2 3 4 3 3 1 5 3 3	158	99,900	65.36	1 6 10 12 15 17 18	148
7	Maximum total project quality	1 1 1 1 1 1 1 1 1 1 1 1 1 1 1 1 1 1	104	168,820	97.59	1 6 9 10 12 15 17 18	168
8		1 1 1 1 1 1 1 2 1 1 1 1 1 1 1 1 1 1	104	168,815	97.47	1 6 9 10 12 15 17 18	147
9		1 1 1 1 1 1 1 3 1 1 1 1 1 1 1 1 1 1	104	168,800	97.39	1 6 9 10 12 15 17 18	181

**Figure 9:**
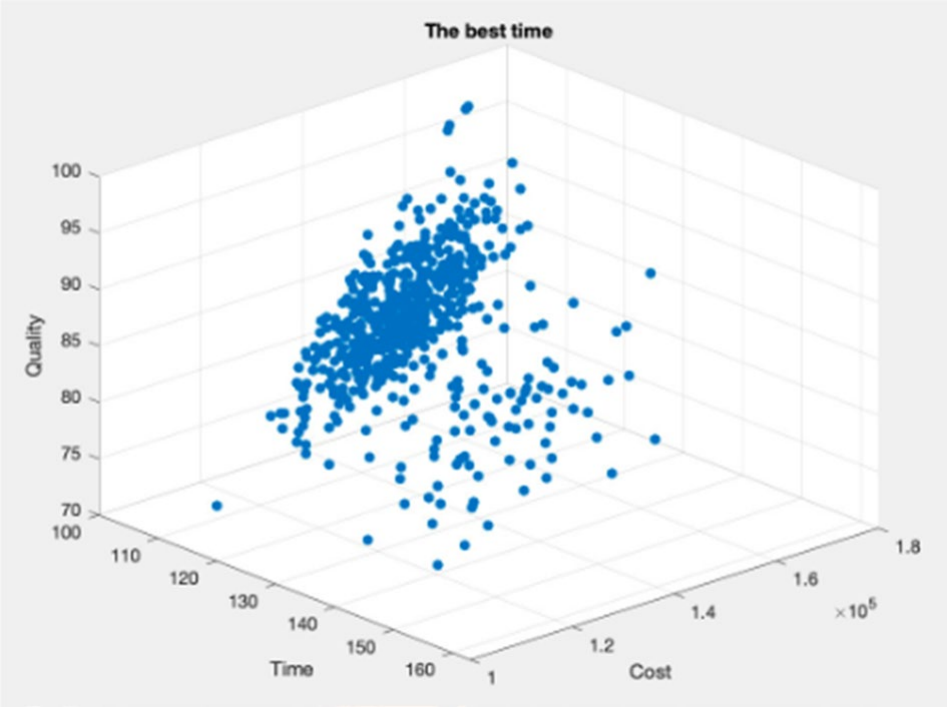
Pareto chart of time–cost–quality optimization obtained using the SMA (optimal time) for case 1.

**Figure 10:**
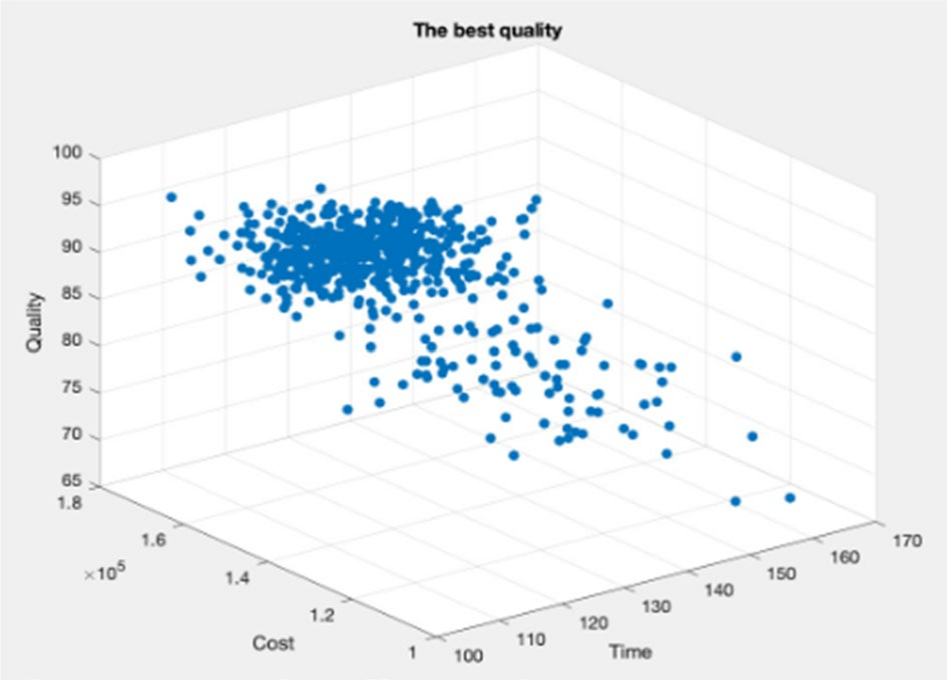
Pareto chart of time–cost–quality optimization obtained using the SMA (optimal quality) case 1.

**Figure 11:**
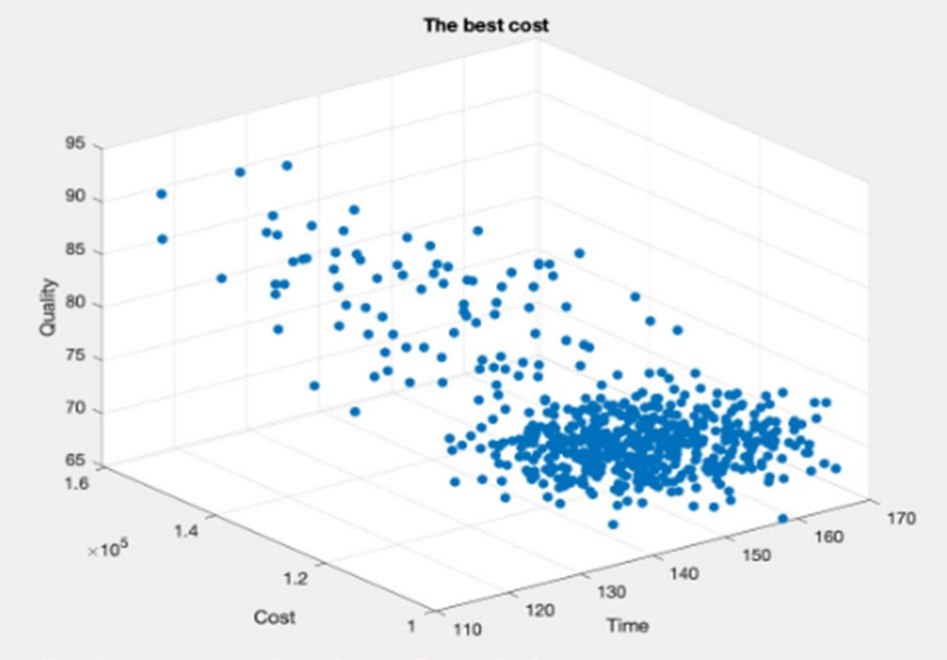
Pareto chart of time–cost–quality optimization obtained using the SMA (optimal cost) case 1.

**Figure 12:**
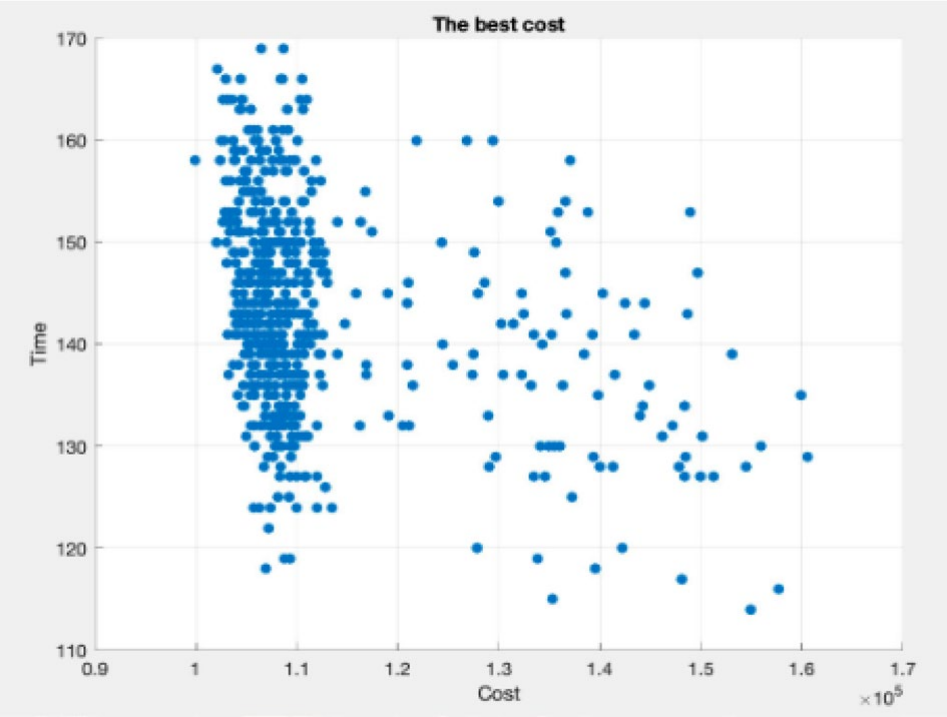
Pareto chart of time–cost optimization obtained using the SMA (optimal cost) case 1.

**Figure 13:**
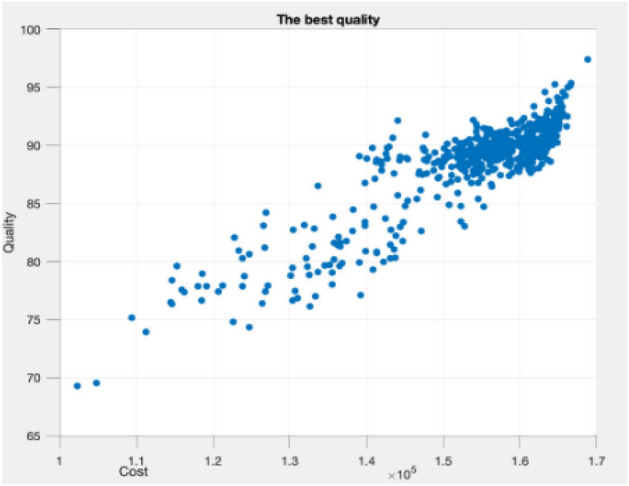
Pareto chart of cost–quality optimization obtained using the SMA (optimal quality) case 1.

**Figure 14:**
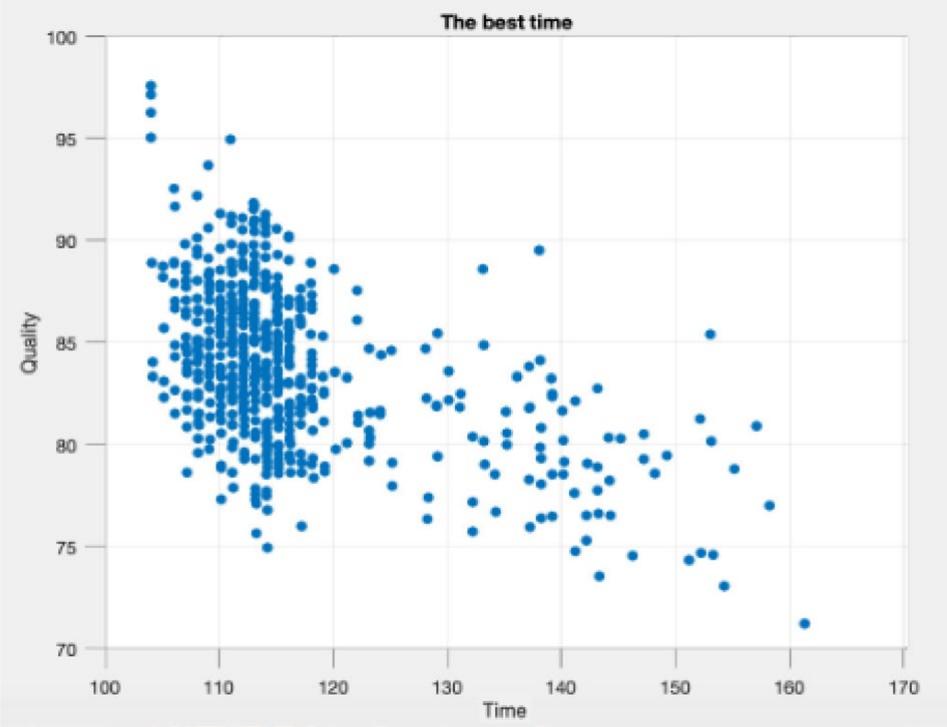
Pareto chart of time–quality optimization obtained using the SMA (optimal time) case 1.

Figures 9, 10, 11, 12, 13, 14 illustrate plots of the Pareto optimization results obtained using the SMA for case 1. These plots illustrate the relationships among the time, cost, and quality of the project. The two-dimensional and three-dimensional visualizations of the relationships among the factors could facilitate a manager’s prediction of performance and potential risks, planning of the appropriate use of resources, and achievement of balanced optimization. The manager could also rely on the actual conditions of time, cost, quality, and related issues to optimize their project activities.

Table 6 displays the best options from the case 2's time–cost-quality and compromised outcome. Additionally, solution 1 shows a low value for time, whereas solution 3 offers the best cost and solution 5 approach for the project's highest quality. Accordingly, the project manager can can choose a solution that balances properties based on the pareto results that have been obtained based on experience and the actual circumstances at the construction site.

**Table 6:** Optimal results for case 2.

Solutions	Partial set	Optimal resource utilization options	Project performance				
			Time (Days)	Cost (Dollars)	Quality (%)	Gant	Iteration (/80)
1	Minimum total project duration	1 1 2 2 1 1 2 1 2 1 1 1 1 1 1 1 1 1 1 1 1 2 1 1 1 1 1 2 1 1 1 1 1 1 1 1 1	172	149,700	77.1861	1 4 7 9 11 13 17 19 26 28 33 34 35 36 37	1
2		1 1 2 2 1 1 2 1 2 1 1 1 1 1 1 1 1 1 1 2 1 1 1 1 1 1 1 2 1 1 1 1 1 1 1 1 1	172	149,300	75.7334	1 4 7 9 11 13 17 19 26 28 33 34 35 36 37	3
3	Minimum total project cost	1 1 1 1 1 1 1 1 1 1 1 1 1 1 1 1 1 1 1 1 1 1 1 1 1 1 1 1 1 1 1 1 1 1 1 1 1	180	145,400	78.5569	1 4 7 9 11 13 17 19 26 28 33 34 35 36 37	10
4		1 1 1 1 1 1 1 1 1 1 1 1 1 1 1 1 1 1 1 2 1 1 1 1 1 1 1 2 1 1 1 1 1 1 1 1 1	189	146,300	76.3713	1 4 7 9 11 13 17 19 26 28 33 34 35 36 37	15
5	Maximum total project quality	1 1 1 1 1 1 1 1 2 1 1 1 1 1 1 1 1 1 1 1 1 1 1 1 1 1 1 1 1 1 1 1 1 1 1 1 1	188	146,000	79.0790	1 4 7 9 11 13 17 19 26 28 33 34 35 36 37	8
6		1 1 2 1 1 1 1 1 2 1 1 1 1 1 1 1 1 1 1 1 1 1 1 1 1 1 1 1 1 1 1 1 1 1 1 1 1	188	147,200	78.8230	1 4 7 9 11 13 17 19 26 28 33 34 35 36 37	9
7	Compromised	1 1 1 1 1 1 1 1 1 1 1 1 1 1 1 1 1 1 1 1 1 1 1 1 1 1 1 1 1 1 1 1 1 1 1 1 1	180	145,400	78.5569	1 4 7 9 11 13 17 19 26 28 33 34 35 36 37	

**Figure 15:**
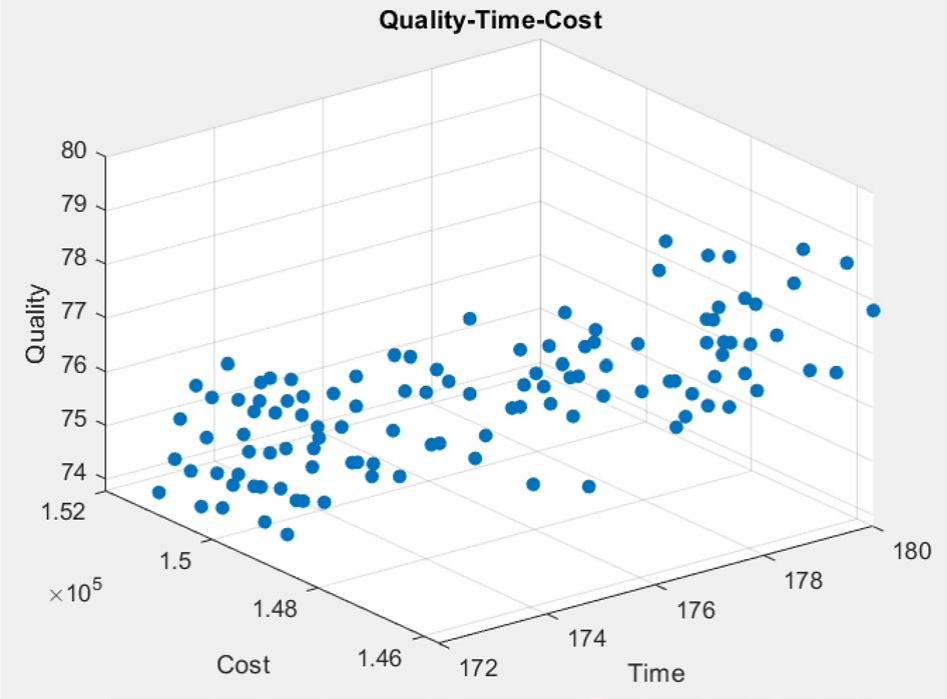
Pareto chart of time–cost–quality optimization obtained using the SMA case 2.

**Figure 16:**
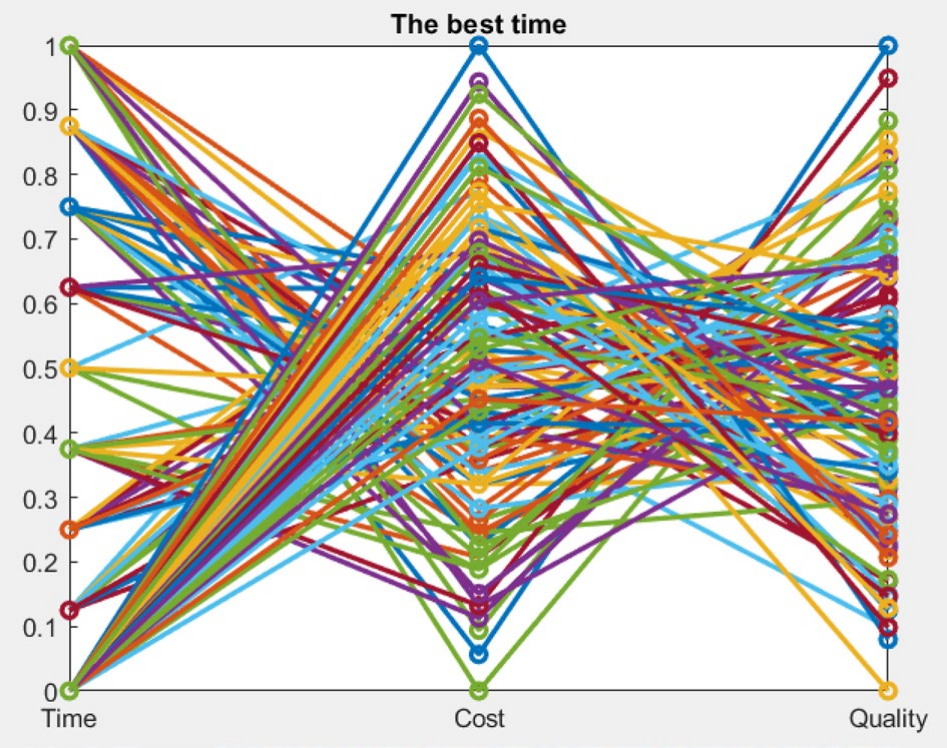
Value path of time–cost-quality optimization obtained using the SMA (optimal time) case 2.

Figures 15, 16 show the pareto outcomes for scenario 2 based on the 3D shape and value path of the Time–Cost–Quality. The project manager can make the appropriate plans to match the current circumstances and the project's external conditions by taking advantage of the three-dimensional space between the three elements. The value path shows the time–cost-quality values by the horizontal axis, the obtained values are connected to form a straight line, while the vertical axis in the path chart shows the values is reduced to the range of [0,1]. The proposed SMA shows that the search for optimal solutions is very good, showing the different transformations between the straight lines of the factors in the search space.

### Comparing the optimization results obtained using the SMA with those obtained using other algorithms

To compare the balanced optimization results, the population and number of iterations of each of the algorithms used in this study were set to 100 and 500, respectively. As listed in Table 7, the project optimization performance achieved by the SMA was higher than that achieved by the other algorithms; the solutions obtained using the SMA were more evenly and widely distributed. Furthermore, the relationships among time, cost, and quality determined using the SMA were clear. Clarifying these relationships can help project managers determine the optimal solution to the problem. In all cases for which solutions were found, the optimization performance of the SMA was superior in terms of convergence compared with those of other algorithms (Table 7). In terms of time, cost, and quality, the SMA was determined to require less time, lower costs, and higher quality. However, to further evaluate the efficiency of the SMA, a few quantitative evaluation indicators of optimization algorithms were used, as discussed in the following section.

**Table 7:** Optimization results obtained using the SMA and other algorithms.

Algorithm	Case	Chain-optimized resources	Project performance		
			Time (day)	Cost (dollars)	Quality (%)
Previous Findings	1	[1.1.1.1.2.1.1.1.1.1.1.1.1.1.1.1.1.1]	104	166,320	95.00
	2	[1.5.3.3.4.3.3.5.1.1.3.1.3.2.1.5.1.1]	114	105,470	71.00
	3	[2.3.1.1.2.3.1.1.1.1.1.1.1.1.1.3.1.1]	115	141,620	90.00
	4	[2.5.1.3.4.2.3.3.1.1.1.1.3.1.1.5.1.1]	109	121,350	77.00
	5	[1.5.1.3.4.3.3.5.1.1.2.1.3.2.1.5.3.1]	124	104,620	72.00
NSGA-II-TCQT	1	[1.1.2.3.1.1.1.1.1.1.1.1.1.1.1.1.1.1]	104	153,320	92.20
	2	[1.2.2.3.1.1.2.1.1.1.2.1.2.1.1.1.1.1]	104	145,820	87.29
	3	[5.3.1.3.4.3.3.2.4.2.1.3.3.2.1.5.3.3]	157	102,915	71.56
	4	[5.4.1.3.4.3.3.1.3.1.1.2.3.1.1.4.3.1]	141	104,850	74.88
	5	[1.1.2.2.1.1.1.1.1.1.1.1.1.1.1.1.1.1]	104	158,320	93.53
MODE-TCQT	1	[1.3.1.1.2.1.1.3.1.1.1.1.1.1.1.5.1.1]	104	163,100	95.10
	2	[1.5.3.3.4.3.3.5.1.1.3.1.3.3.1.5.1.1]	114	105,270	71.55
	3	[1.1.1.2.2.3.1.2.1.1.1.1.1.1.1.2.1.1]	114	133,315	90.06
	4	[2.5.1.3.4.2.3.2.1.1.3.1.3.2.1.5.1.1]	109	120,615	77.01
	5	[5.5.3.3.4.3.3.5.1.1.1.1.3.2.1.5.1.1]	124	104,420	72.08
OMODE-TCQT	1	[1.2.1.1.2.1.1.2.1.1.1.1.1.1.1.2.1.1]	104	164,715	96.17
	2	[1.1.1.2.1.1.1.1.1.1.1.1.1.1.1.1.1.1]	104	158,820	95.03
	3	[1.5.3.3.4.3.3.5.1.1.3.1.3.3.1.5.1.1]	114	105,270	71.55
	4	[5.5.3.3.4.3.3.5.4.2.3.4.3.3.1.5.3.3]	159	99,870	65.24
	5	[1.1.1.1.1.1.1.1.1.1.1.1.1.1.1.2.1.1]	104	167,820	97.33
SMA	1	[1.3.1.1.1.1.1.1.1.1.1.1.1.1.1.2.1.1]	104	166,620	96.77
	2	[1.1.1.1.2.1.1.1.1.1.1.1.1.1.1.3.1.1]	104	165,070	96.28
	3	[5.5.3.3.4.3.3.5.5.2.3.4.3.3.1.5.3.3]	161	99,820	65.15
	4	[5.5.3.3.4.3.3.5.3.2.3.4.3.3.1.5.3.3]	158	99,900	65.36
	5	[1.1.1.1.1.1.1.1.1.1.1.1.1.1.1.1.1.1]	104	168,820	97.59

The benefit of SMA is applied to construction issues, particularly in the management sector of the industrialization business. The outcomes demonstrate that SMA is also applicable to construction management optimization issues in practical settings, with successful and beneficial optimization outcomes. The following list summarizes SMA's successful performance in keeping the model's exploitation and exploration of utility points in balance:

- The algorithm's weight W avoids local optimization during rapid convergence in order to maintain stability and assure quick convergence.

- SMA guarantees that data extraction is accurate and done efficiently early on.

- The decision is improved by making full use of the algorithm's parameter values.

### Comparing the evaluation indicators of the SMA with those of other algorithms

Three issues related to optimization algorithms were considered: convergence of the optimization set, diversity of the optimization set, and wide distribution in the boundary region of the optimization set. Previous studies have used three basic evaluation criteria to assess these issues: accuracy, diversity, and distribution^32^. In this study, these three basic criteria were applied in the context of TCQT.

#### C-metric (C)

C-metric is often used to assess the quality of the true Pareto front of optimization problems. $S_{1} ,S_{2} \subseteq S$ are considered two sets of decision solutions. C-metric represents the mapping between the ordered pair (*S*_*1*_*, S*_*2*_) and the interval [0,1] and is expressed as follows:

11 $$C(S_{1} ,S_{2} ) = \frac{{\left| {\left\{ {a_{2} \in S_{2} ;\exists a_{1} \in S_{1} :a_{1} \le a_{2} } \right\}} \right|}}{{\left| {S_{2} } \right|}}$$

The numerator in Eq. (11) represents the number of solutions in *S*_*2*_, which are dominated by at least one solution in *S*_*1*_; the denominator represents the total solutions in *S*_*2*_. Table 8 presents the C-metric values derived for the five algorithms, where A1, A2, A3, A4, and A5 correspond to the SMA, OMODE, MODE, MOPSO, and NSGA-II, respectively. As indicated in this table, the SMA accounted for more than 49% of the OMODE solution, 59% of the MODE solution, 78% of the MOPSO solution, and 76% of the NSGA-II solution, on average. Table 9 also show A1, A2, A3, A4 and A5 represent for SMA, CAMODE, MODE, MOPSO and NSGA-II. According to the results in Table 9, the SMA is higher more than 12% of the CAMODE, 13% of the MODE, 75% of the MOPSO and 69% of the NSGA-II.

**Table 8:** C-metric values for case 1.

C-metric	C (A1,A2)	C (A2,A1)	C (A1,A3)	C (A3,A1)	C (A1,A4)	C (A4,A1)	C (A1,A5)	C (A5,A1)
Best	0.74	0.1	0.82	0.15	0.90	0.18	0.96	0.10
Worst	0.21	0.02	0.33	0.03	0.64	0.01	0.38	0.00
Average	**0.49**	0.05	**0.59**	0.08	**0.78**	0.11	**0.76**	0.05
Std	0.27	0.04	0.25	0.06	0.13	0.09	0.29	0.05

Significant values are in bold.

**Table 9:** C-metric values for case 2.

C-metric	C (A1,A2)	C (A2,A1)	C (A1,A3)	C (A3,A1)	C (A1,A4)	C (A4,A1)	C (A1,A5)	C (A5,A1)
Best	0.26	0.00	0.38	0.00	0.90	0.02	0.96	0.03
Worst	0.00	0.00	0.00	0.00	0.34	0.00	0.23	0.00
Average	**0.12**	0.00	**0.13**	0.00	**0.75**	0.01	**0.69**	0.01
Std	0.15	0.00	0.18	0.00	0.31	0.01	0.27	0.01

Significant values are in bold.

#### Spread (SP)

This indicator represents the extent of spread achieved among the nondominated solutions and is expressed as follows:

12 $$SP = \frac{{\sum\nolimits_{i = 1}^{k} {d(E_{1} ,\Omega )} + \sum\nolimits_{x \in \Omega } {\left| {d(X,\Omega ) - \mathop d\limits^{ - } } \right|} }}{{\sum\nolimits_{i = 1}^{k} {d(E_{1} ,\Omega ) + (\left| \Omega \right| - k)\mathop d\limits^{ - } } }}$$

where $\Omega$ is a set of solutions and $\left| \Omega \right|$ is the total number of solutions in $\Omega$. (*E*_*1*_*,…,E*_*K*_) are *k* extreme solutions in the true Pareto front, and *k* is the number of objectives. $d(X,\Omega ) = \mathop {\min }\limits_{Y \in \Omega ,Y \ne X} \left| {\left| {F(X) - F(Y)} \right|} \right|$ is the minimum Euclidean distance between solution *X* and its neighboring solutions in the obtained nondominated set $\Omega$. $\mathop d\limits^{ - } = \frac{1}{\Omega }\sum\limits_{X \in \Omega } {d(X,\Omega )}$ is the mean value of $d(X,\Omega )$. A lower *SP* value indicates a better distribution and diversity of nondominated solutions. Tables 10, 11 lists the spread metrics derived for the various algorithms. The SMA was determined to have optimal scores in all metrics.

**Table 10:** Spread metrics for case 1.

Spread	SMA	OMODE	MODE	MOPSO	NSGA-II
Best	0.6790	0.6931	0.7308	0.7043	0.9761
Worst	0.8369	0.8461	1.0670	0.9641	1.2699
Average	**0.7575**	0.7671	0.8688	0.7848	1.1005
Std	0.0789	0.0428	0.1010	0.0957	0.0876

Significant values are in bold.

**Table 11:** Spread metrics for case 2.

Spread	SMA	CAMODE	MODE	MOPSO	NSGA-II
Best	0.5523	0.5714	0.8571	0.5714	0.8571
Worst	1.5561	1.6000	2.1250	2.0000	2.1250
Average	**0.9982**	1.0611	1.5203	1.4057	1.6069
Std	0.4836	0.5172	0.6108	0.6598	0.6486

Significant values are in bold.

#### Hyper volume (HV)

This indicator is used to evaluate the volume (in the objective space) of the members of a nondominated set $\Omega$ for minimizing all objectives of a problem. Mathematically, a hypercube *v*_*i*_ is constructed for each solution $X_{i} \in \Omega$ with reference point *W* and solution *X*_*i*_ as the diagonal corners of the hypercube. *HV* can be expressed as follows:

13 $$HV = \mathop \cup \limits_{i = 1}^{\left| \Omega \right|} v_{i}$$

After normalization, the *HV* values are typically confined to the range [0,1]. Tables 12, 13 presents the *HV* values obtained for each of the five compared algorithms. Similarly, the SMA was determined to have optimal *HV* values.

**Table 12:** HV values for case 1.

HV	SMA	OMODE	MODE	MOPSO	NSGA-II
Best	0.9998	0.9998	0.9775	0.9832	0.9533
Worst	0.9256	0.9165	0.9158	0.9000	0.9000
Average	**0.9622**	0.9579	0.9481	0.9325	0.9262
Std	0.0371	0.0291	0.0242	0.0287	0.0194

Significant values are in bold.

**Table 13:** HV values for case 2.

HV	SMA	CAMODE	MODE	MOPSO	NSGA-II
Best	0.9592	0.9890	0.9534	0.9087	0.9232
Worst	0.9157	0.9260	0.9113	0.9000	0.9064
Average	**0.9662**	0.9567	0.9347	0.9035	0.9151
Std	0.0391	0.0371	0.0173	0.0035	0.0073

Significant values are in bold.

## Conclusion

This study proposed the use of the SMA, which is based on the behavior of the slime mold, to solve the TCQT optimization problem in construction projects. Project management is a top management domain that necessitates the appropriate evaluation of the performance and role of a construction project. The SMA can help project managers obtain optimal results by minimizing time and cost while still achieving the highest possible construction quality; this can lead to positive project outcomes overall. In addition, the study compared the SMA with other algorithms and demonstrated the superiority and effectiveness of the SMA in solving the TCQT problem in large spaces and preventing local optimization. The SMA can also be used to effectively solve optimization problems, balance diversity, and determine convergence in Pareto models. These results can guide the development of future algorithms. However, algorithms always have limited capabilities, and improving algorithms is crucial for the comprehensive development of artificial intelligence systems for human use.

The optimization of time, cost, and quality is critical in construction projects. Projects completed on time with low cost and high quality can contribute to economic development. On the basis of the results of this study, the following conclusions were drawn:

- The SMA is a potential tool for solving the TCQT problem.

- The results obtained using the SMA are superior to those obtained using other algorithms. The SMA-derived optimization results can help managers make better decisions regarding projects.

- The SMA exhibits fast convergence, stability, and a uniform distribution, rendering it more efficient than other algorithms.

## Directions for future research

Comparing SMA's debut to previous algorithms, it demonstrates the capacity to explore and exploit quite successfully. But while this study was being put into practice, local optimization, which was used to simultaneously optimize three objectives, also amply demonstrated the drawbacks of SMA. The authors suggest combining the SMA model with well-known techniques like such as opposition-based learning, tournament selection and other methods to improve the model in a positive way in order to develop the increasingly superior SMA model and operate to handle optimization issues in the construction industry as well as other societal fields. The authors also suggest using the development model to solve additional construction-related issues, such as safety and environmental impact, in order to better serve the objectives of building construction. The focus of this research contains both many benefits and many drawbacks; as a result, the authors will continue to investigate and experiment to expand the model and embrace new aspects to enhance the research paper in the future.

## Data Availability

The Data generated in this research are available from the corresponding author on request.
